# Meta-analysis of sonication prosthetic fluid PCR for diagnosing periprosthetic joint infection

**DOI:** 10.1371/journal.pone.0196418

**Published:** 2018-04-27

**Authors:** Kan Liu, Jun Fu, Baozhan Yu, Wei Sun, Jiying Chen, Libo Hao

**Affiliations:** 1 Department of Orthopedics, General Hospital of Chinese People’s Liberation Army, Beijing, China; 2 Department of Orthopedics, Beijing University of Chinese Medicine Third Affiliated Hospital, Beijing, China; 3 Department of Intensive Care Unit, Nanyuan Hospital, Beijing, China; IIS-Fundacion Jimenez Diaz, SPAIN

## Abstract

Periprosthetic joint infection (PJI) is a catastrophic complication following total joint arthroplasty. Until now, the diagnosis of PJI is still confronted with difficulties, which is characterized by technical limitations. The question of whether sonication fluid PCR can provide high value in the diagnosis of PJI remains unanswered. This meta-analysis included 9 studies that evaluated PCR assays of sonication fluid for the diagnosis of PJI. The pooled sensitivity, specificity, Positive likelihood ratio (PLR), Negative likelihood ratio (NLR) and Diagnostic odds ratio (DOR) were 0.75 (95% confidence interval [CI], 0.71 to 0.81), 0.96 (CI, 0.94 to 0.97), 18.24 (CI, 6.07 to 54.78), 0.27 (CI, 0.20 to 0.36) and 86.97 (CI, 37.08 to 203.97), respectively. The AUC value of the SROC was 0.9244 (standard error, 0.0212). Subgroup analyses showed that use of multiplex PCR and may improve sensitivity and specificity. The results of this meta-analysis showed that PCR of fluid after sonication is reliable and of great value in PJI diagnosis.

## Introduction

The rise in the number of total joint arthroplasty performed worldwide could result in an increasing number of complications, the most catastrophe of these include periprosthetic joint infection (PJI), with an incidence of 1 to 12% [1,2]. PJI poses a significant burden on patients, surgeons, and the healthcare economy. Early treatment requires early detection and identification of the infectious agent. Unfortunately, diagnosis of infection is difficult and challenging in many cases [3].

Several studies have assessed the diagnostic value of PJI, including PCR techniques of implant sonication samples [4–5]. However, the true diagnostic capabilities of these tests remain controversial and inconsistent. Therefore, we performed a meta-analysis to evaluate the detection validity of sonication fluid PCR in the diagnosis of PJI to provide further evidence for its clinical use.

## Material and methods

The methodological approach to evidence searching and synthesis described in this protocol was conducted according to the Cochrane Collaboration’s Diagnostic Test Accuracy methods [6]. In our study, we performed a literature search, screened the studies identified, and evaluated the studies that related to application of sonication fluid PCR in PJI diagnosis.

### Search strategy

We searched electronic databases including PubMed, EMBASE, Cochrane Library, Web of Science, Science Direct and OVID for articles that were published from the time of database inception to June 2017, using the following medical subject headings (MeSH) or keywords: “periprosthetic joint infection OR prosthesis-related infections” “septic loosening”, “aseptic loosening”, “sonication OR sonicate OR ultrasound”, “PCR OR polymerase chain reaction”. We also manually searched the reference lists of eligible studies and review articles. Animal-only studies and studies that do not report data on the diagnostic performance of our target index were excluded.

### Eligibility criteria

Our reviewers independently evaluated the selected studies according to the following inclusion criteria: (1) the study assessed the accuracy of sonication fluid PCR for the diagnosis of PJI compared with the presence of a sinus tract communicating with the prosthesis, the visible purulence of the synovial fluid or surgical site, simultaneously obtained microbiological cultures from at least two periprosthetic tissue samples or acute inflammation in the histopathological periprosthetic tissue sections; (2) sufficient data were reported to allow the calculation of true-positive (TP), true-negative (TN), false-positive (FP), and false-negative (FN) values; (3) the study reported at least 10 patients, from which data extraction using our standardized data collection form. Discrepancies were resolved by discussion with other reviewers and reanalysis of the original articles.

### Quality assessment

Two reviewers independently screened the retrieved clinical studies for inclusion, extracted data from all included studies and conducted the quality assessment. The methodological quality of the selected studies was evaluated by using the quality assessment of diagnostic accuracy studies tool (QUADAS-2) [7], which was specifically developed for systematic reviews focusing on diagnostic accuracy. When confronted with disagreements, a third reviewer adjudicated.

### Data extraction

Data were extracted independently by two reviewers with all outcomes and then verified by the other reviewers. The following information were abstracted: (1) study characteristics including author, year of publication, country, sample size, study design, sample site and diagnostic criteria; (2) intervention characteristics including ultrasonic conditions, sample conditions, type of PCR and target gene; (3) diagnostic outcomes including sensitivity and specificity, positive likelihood ratio (PLR), and negative likelihood ratio (NLR).

### Statistical analysis

For the analysis of diagnostic value of sonication fluid PCR, all statistical analyses were conducted using Meta-Disc software (version 1.4, Unit of Clinical Biostatistics team, Madrid, Spain). The specificity, sensitivity, PLR, NLR, diagnostic odds ratio (DOR), and area under the curve (AUC) of summary receiver operating characteristic (SROC) were estimated. Meta-regression and subgroup analyses were performed to assess potential heterogeneity. The percentage of the total variation across studies was described by the *I*^2^ statistic, which indicated the existence of significant heterogeneity when the value exceeded 50%. The value of *I*^2^ ranges from 0 to 100%, with 0 implying no observed heterogeneity, and larger values indicating increasing heterogeneity [8]. Analysis of heterogeneity between studies was conducted using the *χ*^2^ test. If there was no significant heterogeneity between studies (*P*>0.1, *I*^2^≤50%), the analysis was performed using a fixed-effects model; otherwise, the random effects model (*P≤*0.1, *I*^2^>50%) was used [9].

## Results

**Fig 1:**
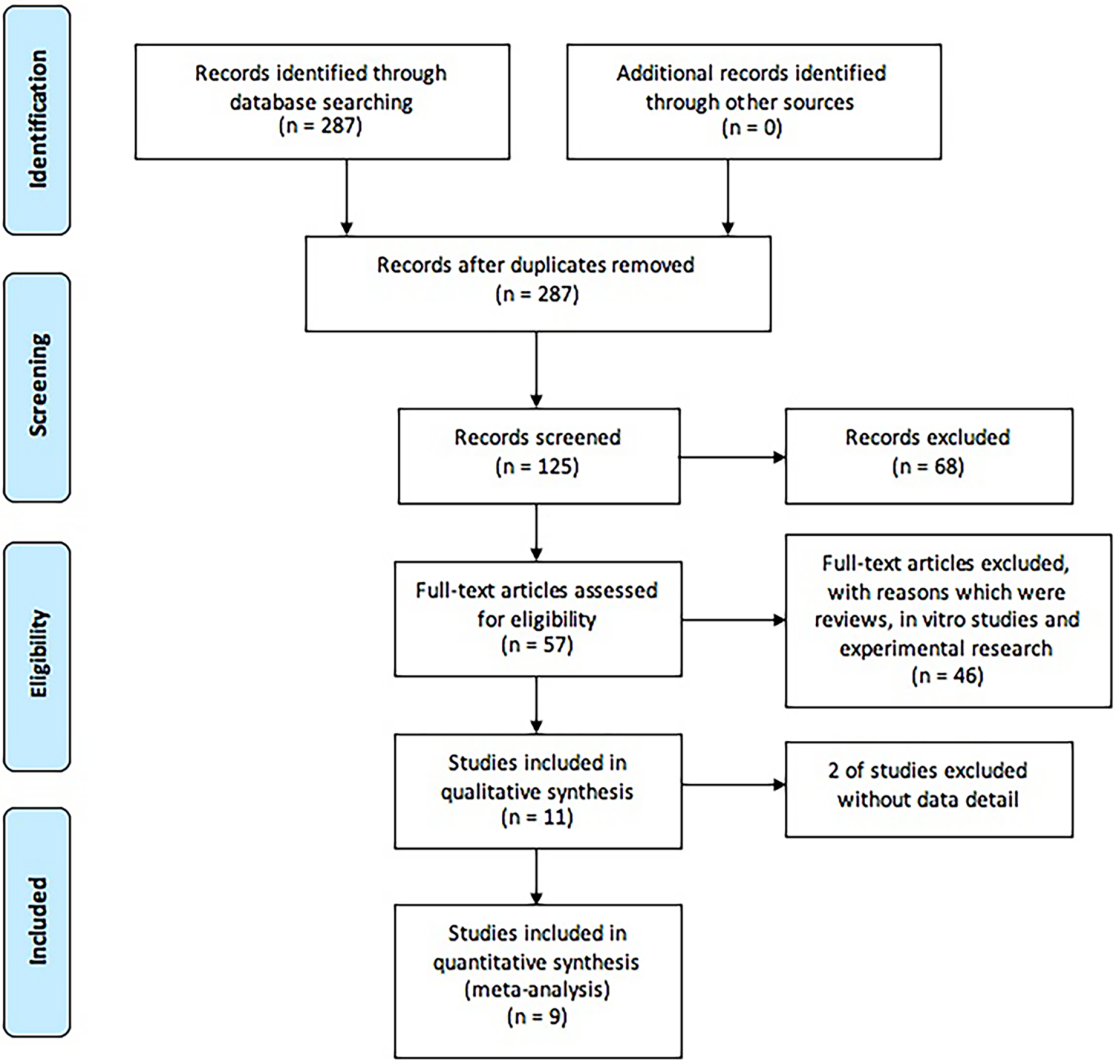
Flow diagram of the selection process for eligible studies.

**Fig 2:**
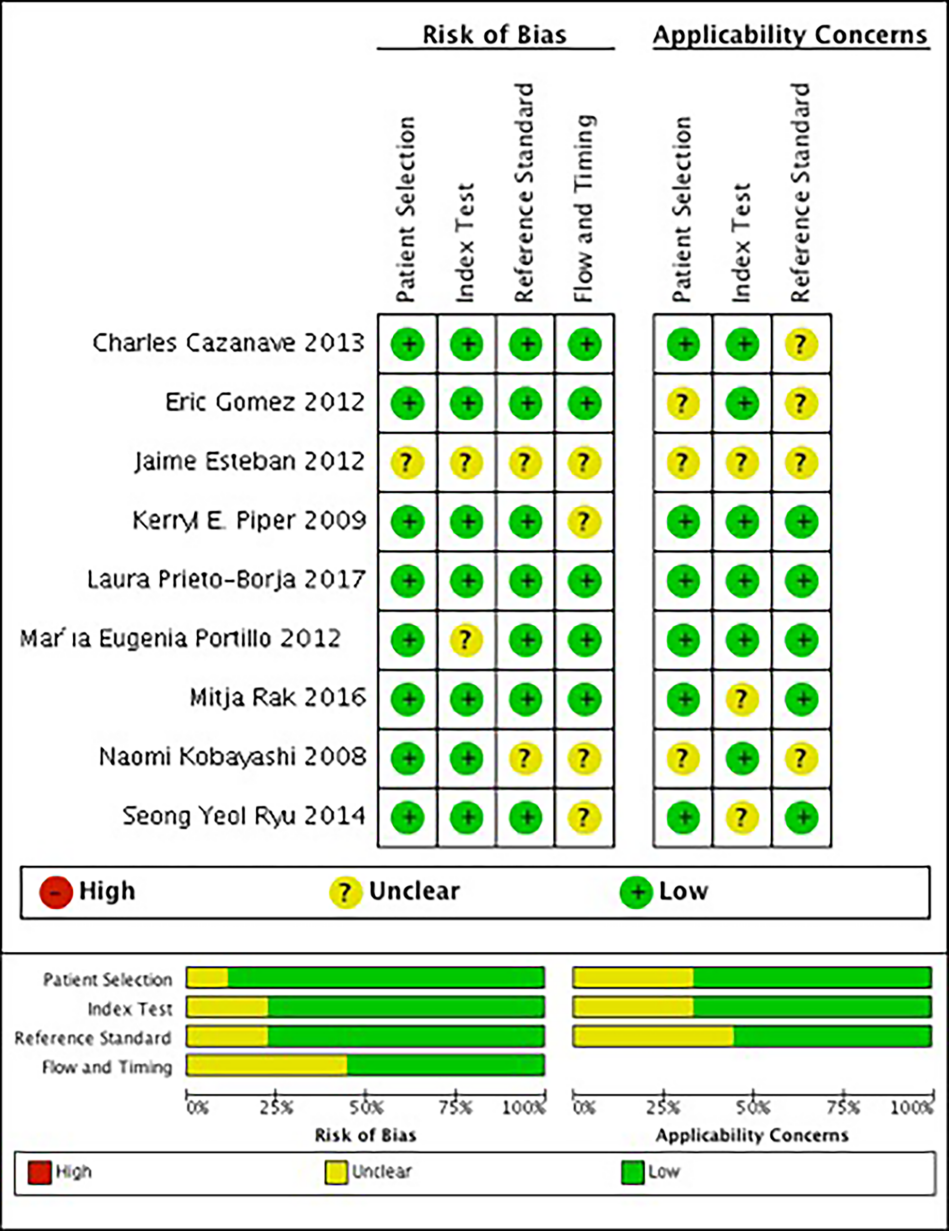
Methodological quality assessment of included studies.

Of the yielded 287 primary articles, 162 of which were excluded with the reasons of duplicates. Among the left 125 articles, 114 were excluded after reviewing the title, abstract and full text of the articles. After reading the whole 11 articles included, 2 were unqualified due to insufficient data. Finally, a total of 9 studies were considered suitable for the diagnostic meta-analysis [5, 10–17], the flow diagram is shown in Fig 1. Graphical summary of the methodological assessment based on QUADAS-2 quality assessment for the recruited studies of meta‑analysis is illustrated in Fig 2. All of which were of moderate to high quality. Detailed characteristics of individual study are summarized in Table 1.

**Table 1:** Characteristics of included studies for meta-analysis.

Study	Country	No. of patients	Mean age (years)	Study design	Vortexing	Centrifugation	Sample condition	Sample site(s)	PCR type	Target gene	Diagnostic standard
**Kobayashi 2008**	Japan	52	NA	Prospective	No	Yes	Fresh	Hip, knee	RT S- and BR-PCR	S probes, 16S rRNA gene	M
**Piper 2009**	USA	136	65	Retrospective	Yes	Yes	Frozen	Shoulder	RT-qPCR	16S rRNA gene	IOF, H
**Portillo 2012**	Spain	86	73	Prospective	Yes	No	Fresh	Hip, knee, elbow, shoulder	RT multiplex PCR	NA	IOF, H, M
**Esteban 2012**	Spain	75	66	Retrospective	No	Yes	Frozen	Hip, knee	RT-PCR	16S rRNA gene	IOF, M
**Gomez 2012**	USA	366	66	Retrospective	Yes	Yes	Frozen	Hip, knee	RT-qPCR	16S rRNA gene	IOF, H
**Cazanave 2013**	USA	434	67	Retrospective	Yes	Yes	Fresh	Hip, knee	RT Multiplex PCR	NA	IOF, H
**Ryu 2014**	USA	36	67	Retrospective	Yes	Yes	Frozen	Knee	RT Multiplex PCR	NA	IOF, H, M
**Rak 2016**	Slovenia	87	70	Prospective	Yes	Yes	Fresh	Hip, knee	RT BR-PCR	16S rRNA gene	MSIS
**Prieto-Borja 2017**	Spain	68	73	Prospective	No	No	Frozen	Hip, knee, shoulder	RT Multiplex PCR	NA	IDSA

RT, real time; BR, broad-range; qPCR, quantitative PCR; S, Staphylococcus; H, histological examination; IOF, intraoperative finding; M, microbiological or laboratory examination; MSIS: Musculoskeletal Infection Society; IDSA, Infectious Diseases Society of America; NA, not available.

**Fig 3:**
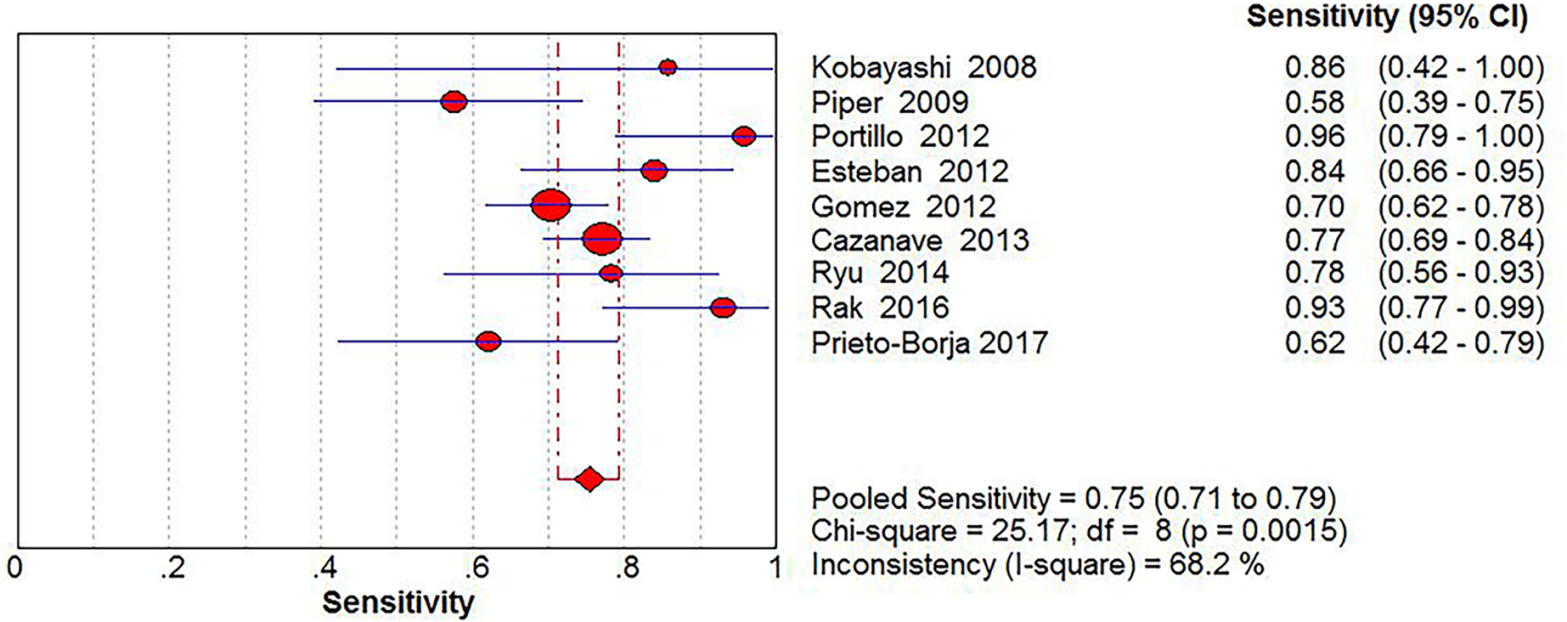
Forest plots of sensitivity of sonication fluid PCR for PJI diagnosis.

**Fig 4:**
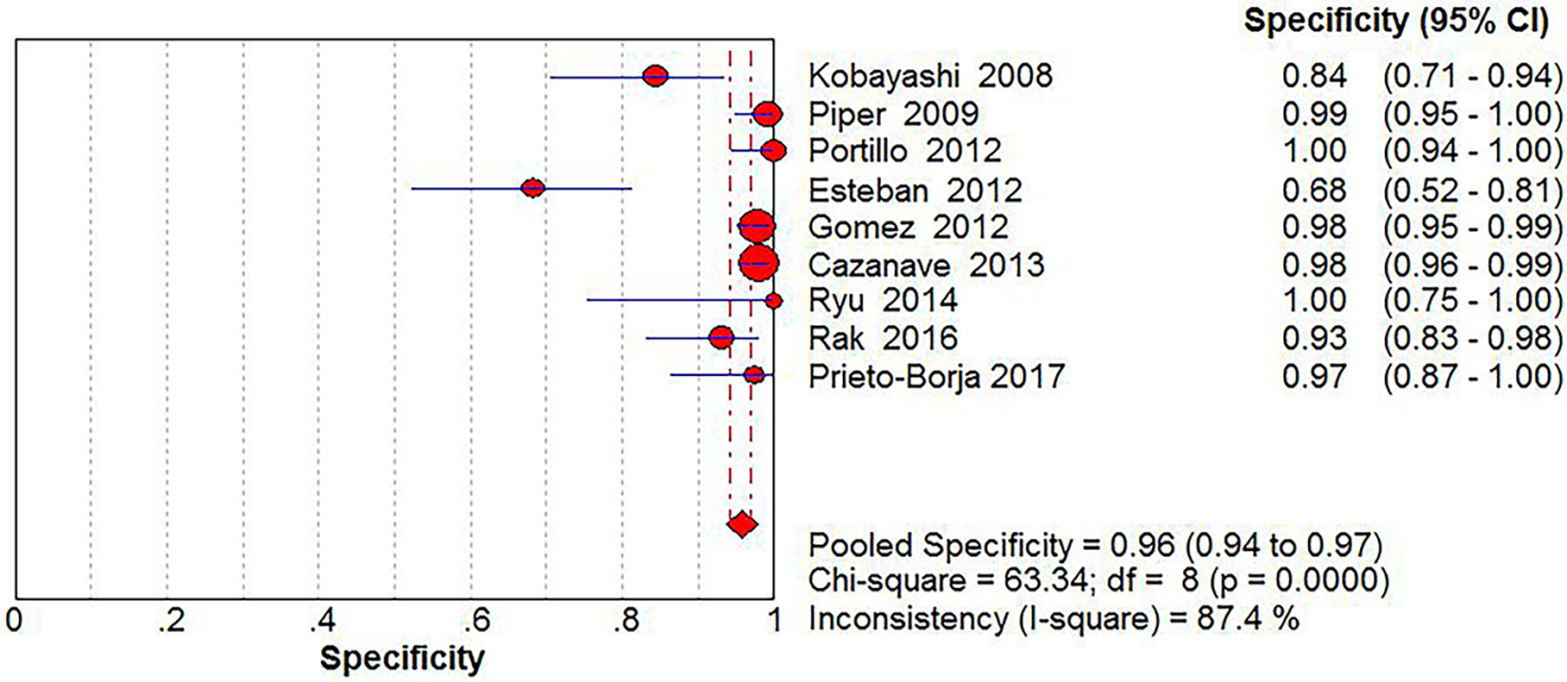
Forest plots of specificity of sonication fluid PCR for PJI diagnosis.

**Fig 5:**
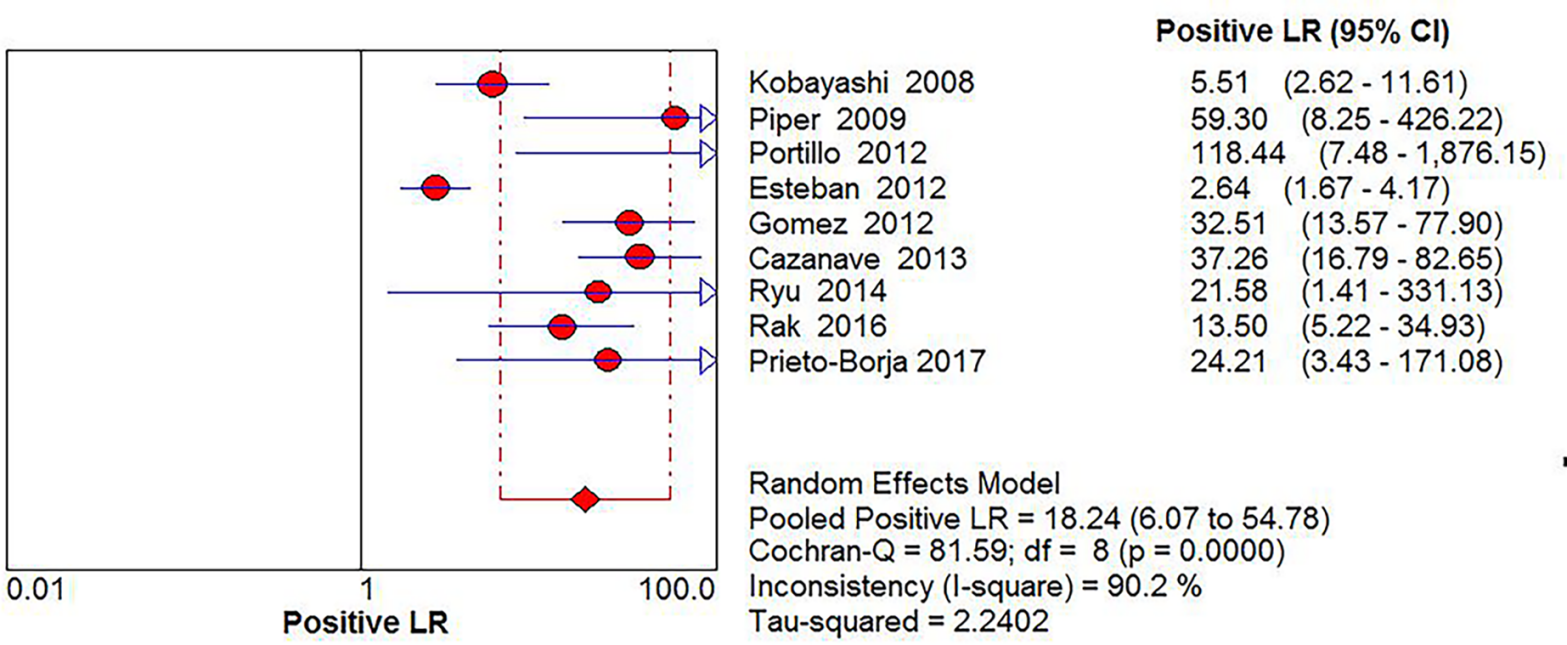
Forest plots of positive likelihood ratio of sonication fluid PCR for PJI diagnosis.

**Fig 6:**
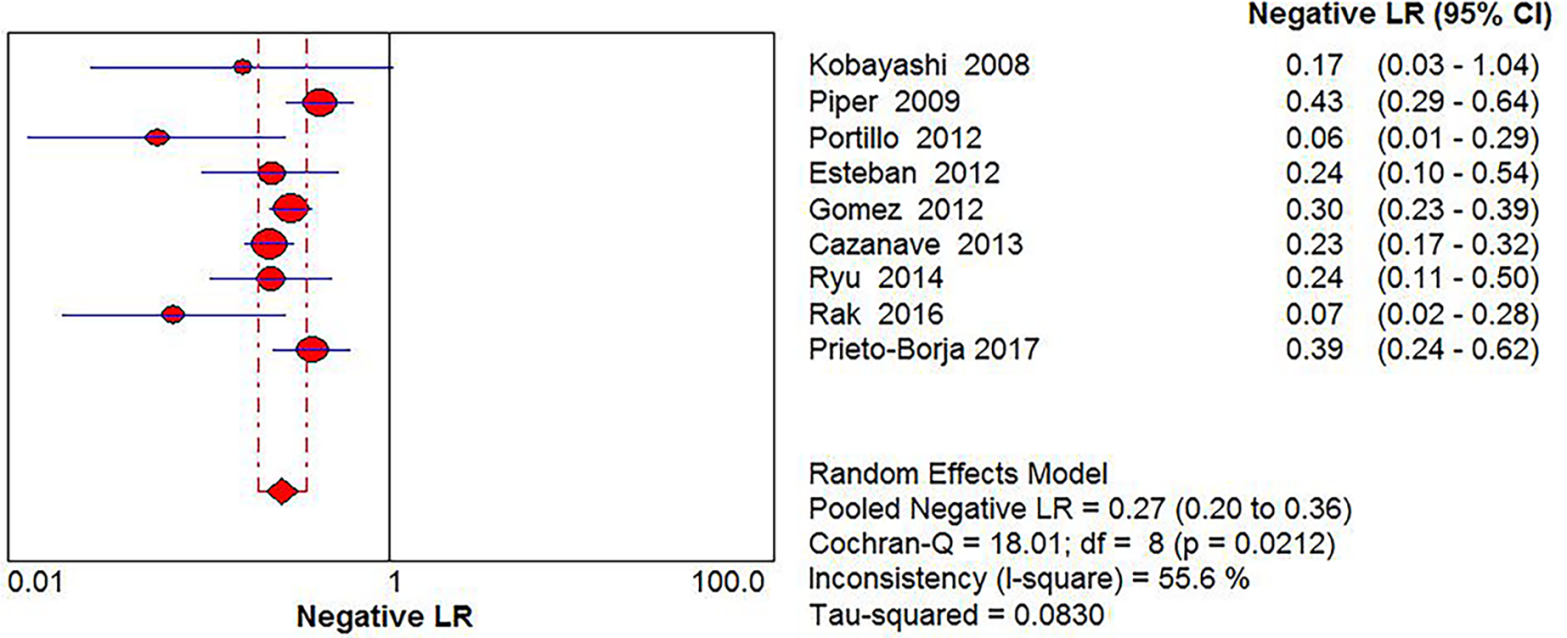
Forest plots of negative likelihood ratio of sonication fluid PCR for PJI diagnosis.

**Fig 7:**
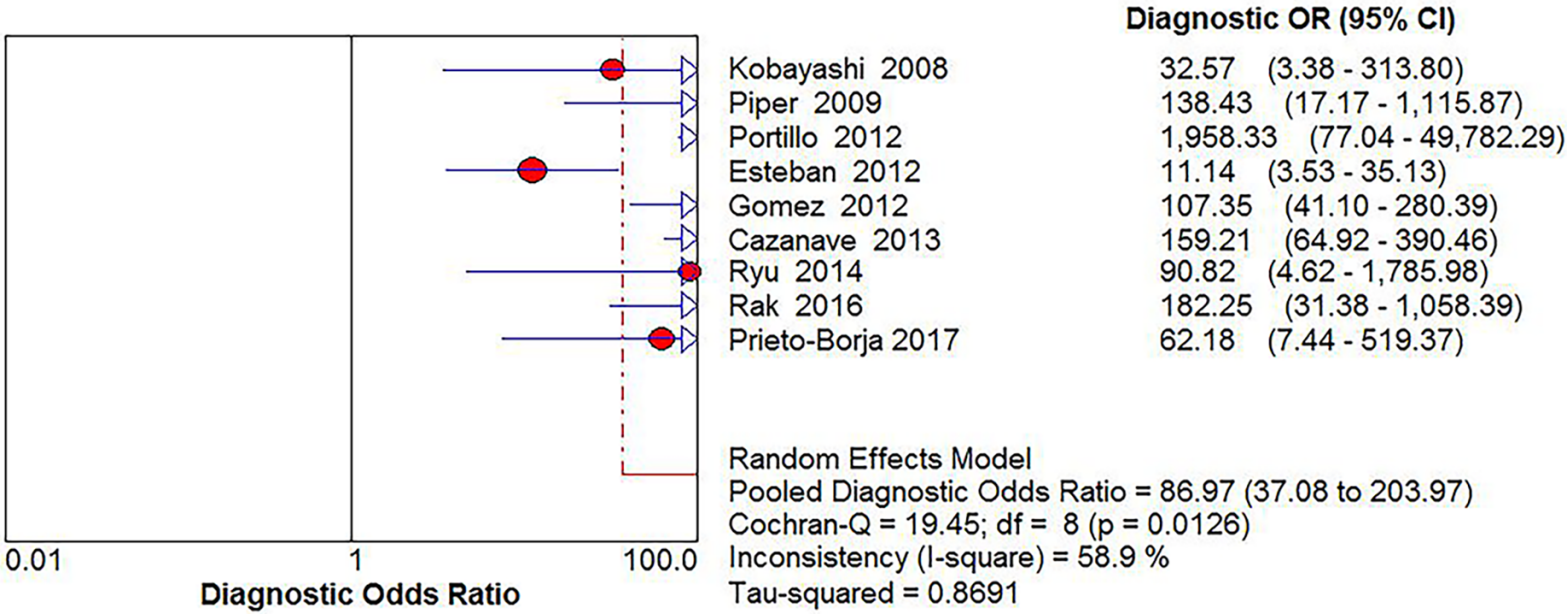
Forest plots of diagnostic odds ratio of sonication fluid PCR for PJI diagnosis.

**Fig 8:**
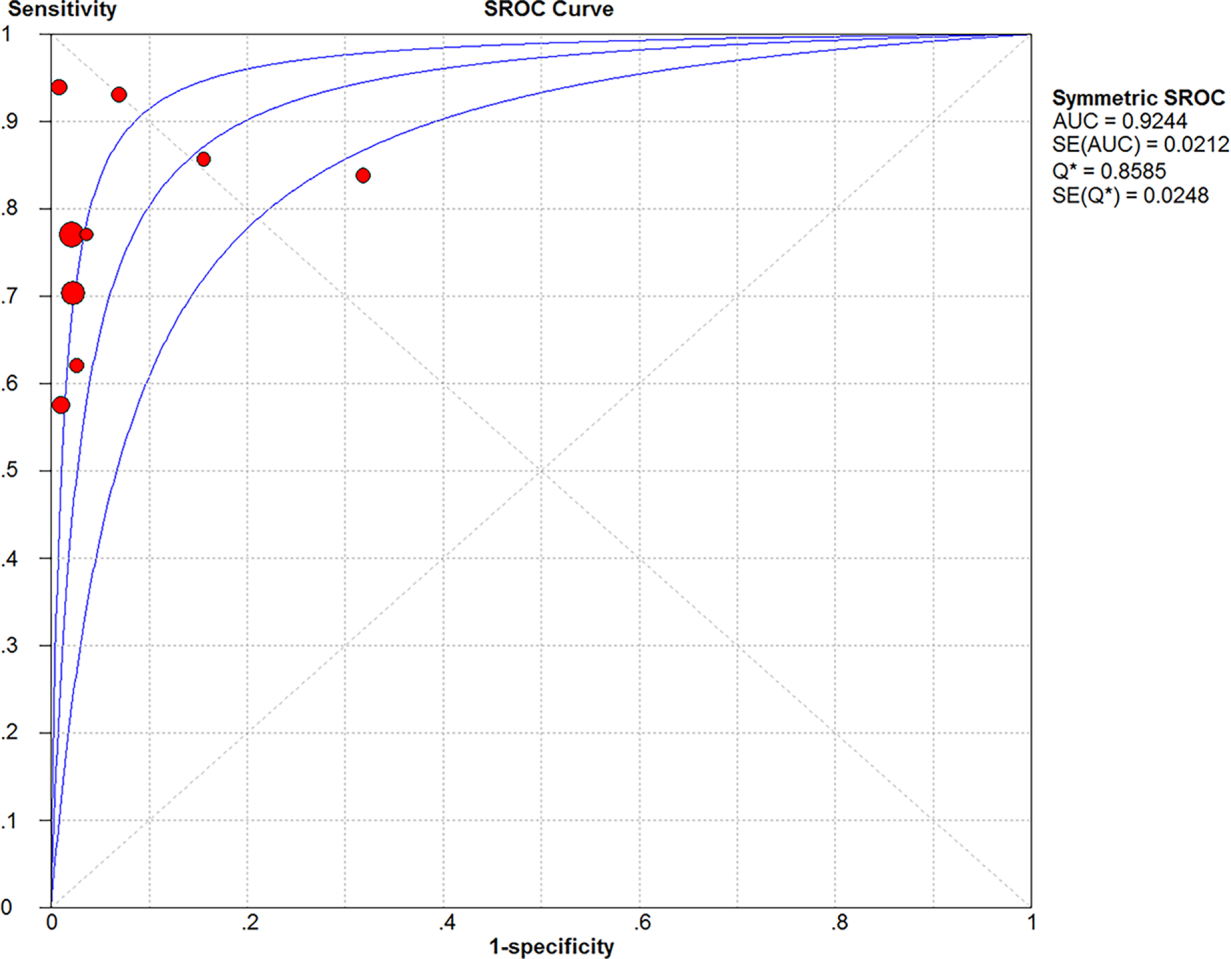
Summary of SROC of sonication fluid PCR for PJI diagnosis.

Significant Heterogeneity was found in sensitivity (*I*^2^ = 68.2%), specificity (*I*^2^ = 87.4%), PLR (*I*^2^ = 90.2%), NLR (*I*^2^ = 55.6%) and DOR (*I*^2^ = 58.9%), respectively; thus, the random-effects model was used. No threshold effect existed (Spearman correlation coefficient: 0.243, *P* = 0.529) in the pooled data. The pooled sensitivity, specificity, PLR, NLR, DOR estimates for the detection of PJI using sonication fluid PCR were 0.75 (95% confidence interval [CI], 0.71 to 0.79), 0.96 (CI, 0.94 to 0.97), 18.24 (CI, 6.07 to 54.78), 0.27 (CI, 0.20 to 0.36), and 86.97(CI, 37.08 to 203.97), respectively (Figs 3, 4, 5, 6 and 7). The SROC plot showed the summary sensitivity and specificity and the 95% confidence and prediction regions, with an AUC of 0.9244 (standard error 0.0212) (Fig 8).

**Fig 9:**
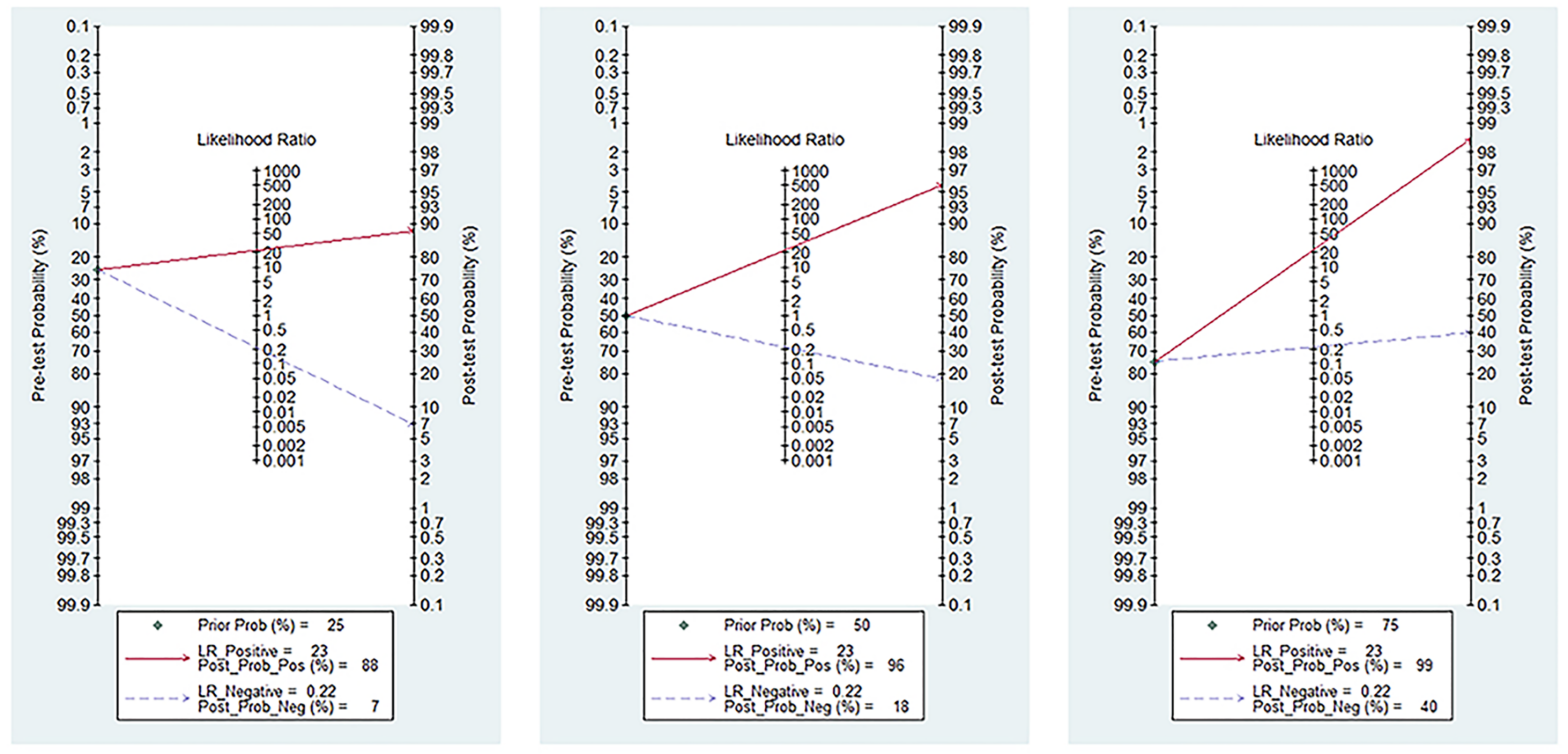
Pre-test probabilities and likelihood ratios (LR) for sonication fluid PCR.

We used the likelihood ratios to simulate low, moderate, and high clinical scenarios using 25%, 50%, and 75% pre-test probabilities of PJI and further calculated and plotted post-test probability on Fagan nomograms (Fig 9). A positive sonication fluid PCR resulted in post-test probabilities of 88%, 96%, and 99%, respectively, and a negative PCR resulted in post-test probabilities of 7%, 18%, and 40%, respectively.

**Fig 10:**
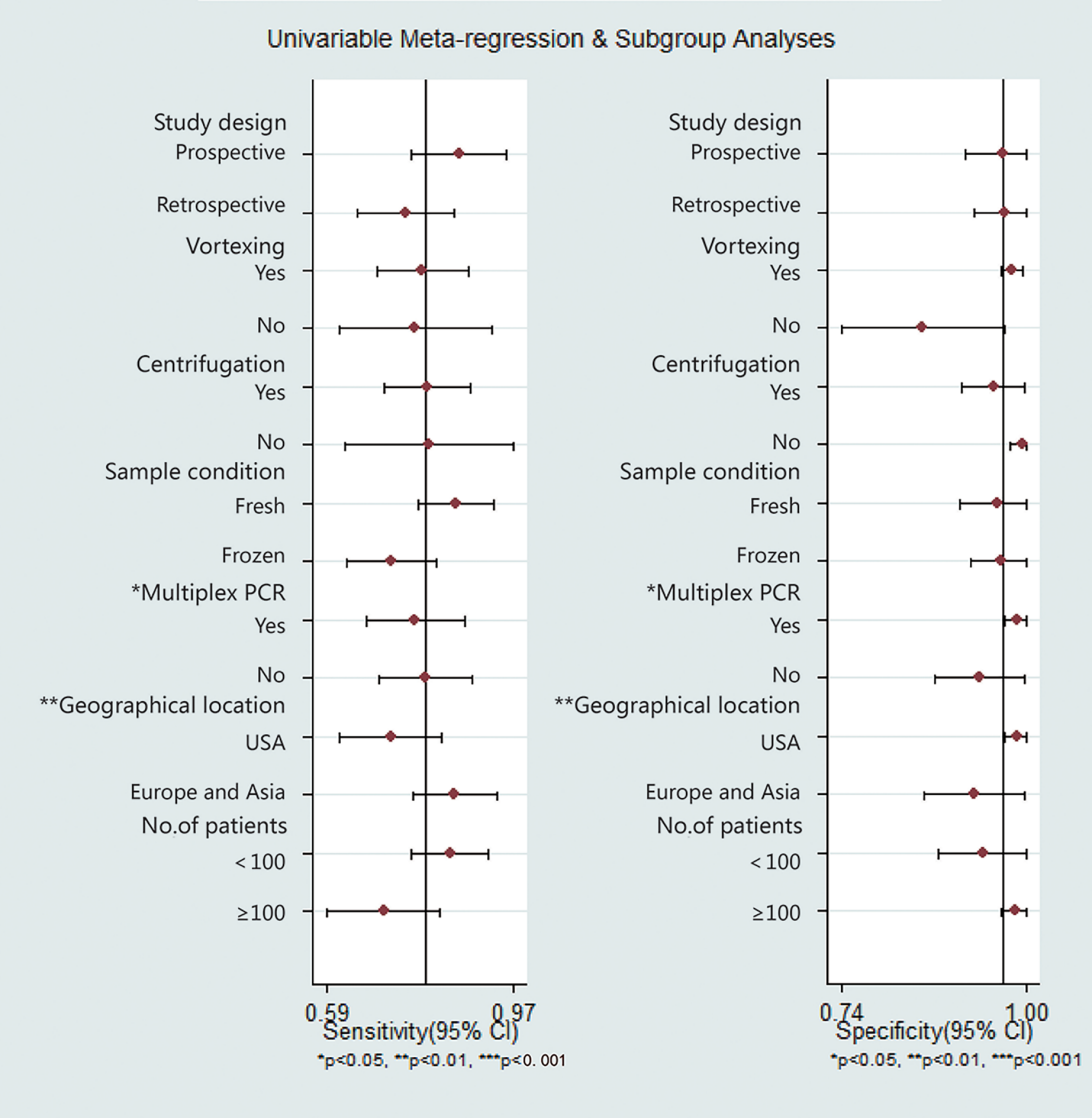
Forest plots of subgroup analyses of sensitivity and specificity.

In subgroup analyses, the test performances varied by study design, vortexing, centrifugation, sample conditions, type of PCR, geographical location, and sample size (Table 2, Fig 10). Compared with non-multiplex PCR, multiplex PCR had a higher specificity of 0.98 (CI, 0.96 to 0.99) (*P*<0.05). Compared to the article of USA, the article of Europe and Asia had a higher sensitivity of 0.83 (CI, 0.75 to 0.90) (*P*<0.05). The sensitivity and specificity of the fresh samples were 0.82 (CI, 0.76 to 0.87) and 0.95 (CI, 0.93 to 0.97), and those of the frozen samples were 0.70 (CI, 0.64 to 0.76) and 0.96 (CI, 0.94 to 0.98), respectively.

**Table 2:** Summary results of subgroup analysis.

Subgroup analyses	No. of studies	No. of patients	Estimates (95% CI)					SROC(SE)
			Sen	Spe	PLR	NLR	DOR	
**Overall studies**	9	1340	0.75(0.71–0.79)	0.96(0.94–0.97)	18.24(6.07–54.78)	0.27(0.20–0.36)	86.97(37.08–203.97)	0.9244(0.0212)
**Study design**								
Prospective	4	293	0.83(0.74–0.90)	0.94(0.90–0.97)	15.27(4.13–56.46)	0.14(0.04–0.56)	126.73(30.89–519.87)	0.9652(0.0304)
Retrospective	5	1047	0.73(0.69–0.78)	0.96(0.94–0.97)	19.82(3.26–120.52)	0.29(0.23–0.37)	71.30(22.90–222.04)	0.8931(0.0308)
**Vortexing**								
Yes	6	1145	0.76(0.71–0.80)	0.98(0.97–0.99)	29.17(18.22–46.71)	0.25(0.17–0.36)	147.33(83.40–260.26)	0.9790(0.0131)
No	3	195	0.75(0.63–0.84)	0.83(0.75–0.89)	5.06(1.91–13.46)	0.33(0.22–0.50)	19.72(7.04–55.20)	0.8804(0.0357)
**Centrifugation**								
Yes	7	1254	0.74(0.70–0.78)	0.95(0.94–0.97)	15.63(5.12–47.77)	0.29(0.22–0.37)	73.17(32.36–165.46)	0.9196(0.0213)
No	2	86	0.77(0.64–0.88)	0.99(0.95–1.00)	41.13(8.34–202.90)	0.17(0.02–1.79)	278.96(9.69–8034.22)	NA
**Sample condition**								
Fresh	4	659	0.82(0.76–0.87)	0.95(0.93–0.97)	18.74(4.89–71.92)	0.14(0.06–0.31)	158.74(56.75–444.04)	0.9643(0.0186)
Frozen	5	681	0.70(0.64–0.76)	0.96(0.94–0.98)	17.91(2.65–121.29)	0.33(0.27–0.40)	54.77(17.02–176.28)	0.8827(0.0342)
**Multiplex PCR**								
Yes	4	624	0.74(0.67–0.79)	0.98(0.96–0.99)	36.65(18.39–73.06)	0.25(0.15–0.40)	156.34(65.58–372.73)	0.9990(0.0023)
No	5	716	0.77(0.71–0.83)	0.94(0.91–0.96)	11.63(3.13–43.22)	0.28(0.19–0.43)	59.53(18.29–193.77)	0.9228(0.0287)
**Geographical location**								
USA	4	972	0.73(0.67–0.77)	0.98(0.97–0.99)	35.78(20.59–62.18)	0.30(0.23–0.39)	130.89(70.97–241.38)	0.9720(0.0406)
Europe and Asia	5	368	0.83(0.75–0.90)	0.90(0.85–0.93)	9.80(3.03–31.68)	0.17(0.07–0.41)	71.09(14.33–352.69)	0.9377(0.0364)
**No. of patients**								
<100	6	404	0.83(0.75–0.88)	0.90(0.86–0.93)	10.67(3.46–32.90)	0.19(0.10–0.37)	71.11(17.47–289.43)	0.9343(0.0321)
≥100	3	936	0.72(0.67–0.77)	0.98(0.97–0.99)	36.56(20.80–64.28)	0.31(0.22–0.42)	133.01(71.17–248.59)	0.9762(0.0376)

Sen, Sensitivity; Spe, Specificity; PLR, Positive likelihood ratio; NLR, Negative likelihood ratio; DOR, Diagnostic odds ratio; SROC, Summarized receiver-operating curve; CI, Confidence interval; SE, Standard error; NA, Not available.

## Discussion

PJI is currently one of the most catastrophic complications associated with TJA. Since PJI diagnosis remains a challenge, many preoperative and intraoperative tests have been employed [1–4, 18]. Unfortunately, the current diagnostic methods are not highly accurate. Historically, intraoperative tissue culture has been used as the gold standard in most hospitals, although the results of culture lack optimal sensitivity (range, 0.70 to 0.90) or specificity (range, 0.67 to 0.91) and are sometimes difficult to interpret, especially when few samples are analyzed [3, 19–23].

In fact, the true diagnostic ability of cultures depends on the accurate recovery of bacteria from samples. Dislodging bacteria from the prosthetic surface by sonication may be an appropriate option [24]. In addition, Zhai et al. found that sonication fluid cultures (SFC) had a high sensitivity and a very high specificity for diagnosing PJI [25]. Further, various factors may influence the diagnostic accuracy of sonication prosthetic fluid samples. Most studies considered false-positive results to be caused by specimen contamination and false-negative results to be caused by prior antibiotic treatment, which may have induced an underestimated sensitivity [24, 25].

PCR techniques have demonstrated beneficial diagnostic value for diagnosing PJI in recent years. Compared to intraoperative tissue culture, PCR theoretically has higher sensitivity, a faster turnaround time, and is not as affected by antibiotic treatments [14]. However, differences in sample types analyzed by PCR may influence the diagnostic ability of PJI. Sonication prosthetic fluid samples may offer additional insight for improving the diagnostic accuracy of PCRs for diagnosing PJI [5, 12–14]. Guidelines for PJI by the American Academy of Orthopaedic Surgeons and the Infectious Diseases Society of America recommend further “high evidence”-based studies to evaluate the diagnostic value of PCR [18].

Our results showed that PCR is another diagnostic method that has an equivalent or better diagnostic value to that of intraoperative tissue culture and may add important insight into the diagnosis of PJI. However, the main problems in the diagnosis of PJI are recovery and identification of bacteria from the samples. Whether relying on intraoperative tissue culture or PCR, the bacterial recovery from the samples is always one of the most important aspects in the diagnosis of PJI. In this meta-analysis, sonication prosthetic fluid samples for PCR had an adequate diagnostic value for the detection of PJI. It was estimated that, in current practice, the sensitivity and specificity of PCR are approximately 75% and 96%, respectively. Our subgroup analyses showed that compared with non-multiplex PCR, use of multiplex PCR had a higher specificity (0.98 versus 0.94, *P*<0.05). However, this type of PCR cannot satisfy both increased sensitivity and increased specificity concurrently.

Moreover, the number of samples taken for PCR may impact the diagnostic sensitivity and specificity of PCR. Marin et al. [22] showed that when only considering the number of positive samples, a PCR-positive result in one sample had good specificity and a positive predictive value for PJI (specificity, 0.96; positive predictive value, 0.92). The best combination of results for PCR was observed when 5 samples were studied and the same microorganism was detected in 2 of them (sensitivity, 0.94; specificity, 1.00). In addition, in our meta-analysis, there were 112 false-negative results from 9 studies. Most of the included studies explained that the false-negative resulted from the patient receiving antibiotics previous to sampling [5, 11–14].

This study had some limitations. First, there was no established gold standard for diagnosing PJI, which is a universal drawback to all studies assessing PCR procedures for diagnostic accuracy in the detection of PJI. In this meta-analysis, individual studies cited different reference standards. Misclassification bias, which results from the use of an imperfect reference standard, may affect the estimates of diagnostic accuracy of a tested method and lead to underestimated diagnostic accuracy. Second, not all studies explicitly stated whether they were performed prospectively, which may reduce the strength of our study conclusions. We performed subgroup analysis and examined study design as possible sources of heterogeneity. Third, despite the summary results of this meta-analysis had high statistical heterogeneity, which may have led to an overestimation of the true diagnostic performance, a number of the significant differences in the subgroup analyses are based on only two sources including type of PCR and Geographical location.

## Conclusions

In summary, PCR for sonication prosthetic fluid was found to have adequate and clinically acceptable diagnostic values for detecting PJI, with a sensitivity of 75% and specificity of 96%. Future cost-effectiveness of this test studies should be performed.

## Supporting information

S1 PRISMA Checklist PRISMA checklist. (DOC)
